# Impaired Transcriptional Activity of Nrf2 in Age-Related Myocardial Oxidative Stress Is Reversible by Moderate Exercise Training

**DOI:** 10.1371/journal.pone.0045697

**Published:** 2012-09-24

**Authors:** Sellamuthu S. Gounder, Sankaranarayanan Kannan, Dinesh Devadoss, Corey J. Miller, Kevin S. Whitehead, Shannon J. Odelberg, Matthew A. Firpo, Robert Paine, John R. Hoidal, E. Dale Abel, Namakkal S. Rajasekaran

**Affiliations:** 1 Divisions of Cardiology and Pulmonary, Department of Internal Medicine, University of Utah Health Sciences Center, Salt Lake City, Utah, United States of America; 2 Department of Pediatric Research, University of Texas M. D. Anderson Cancer Center, Houston, Texas, United States of America; 3 Department of General Surgery, University of Utah Health Sciences Center, Salt Lake City, Utah, United States of America; 4 Division of Pulmonary, Department of Internal Medicine, University of Utah Health Sciences Center, Salt Lake City, Utah, United States of America; 5 Division of Endocrinology, Department of Internal Medicine, University of Utah Health Sciences Center, Salt Lake City, Utah, United States of America; The Chinese University of Hong Kong, Hong Kong

## Abstract

Aging promotes accumulation of reactive oxygen/nitrogen species (ROS/RNS) in cardiomyocytes, which leads to contractile dysfunction and cardiac abnormalities. These changes may contribute to increased cardiovascular disease in the elderly. Inducible antioxidant pathways are regulated by nuclear erythroid 2 p45-related factor 2 (Nrf2) through antioxidant response cis-elements (AREs) and are impaired in the aging heart. Whereas acute exercise stress (AES) activates Nrf2 signaling and promotes myocardial antioxidant function in young mice (∼2 months), aging mouse (>23 months) hearts exhibit significant oxidative stress as compared to those of the young. The purpose of this study was to investigate age-dependent regulation of Nrf2-antioxidant mechanisms and redox homeostasis in mouse hearts and the impact of exercise. Old mice were highly susceptible to oxidative stress following high endurance exercise stress (EES), but demonstrated increased adaptive redox homeostasis after moderate exercise training (MET; 10m/min, for 45 min/day) for ∼6 weeks. Following EES, transcription and protein levels for most of the ARE-antioxidants were increased in young mice but their induction was blunted in aging mice. In contrast, 6-weeks of chronic MET promoted nuclear levels of Nrf2 along with its target antioxidants in the aging heart to near normal levels as seen in young mice. These observations suggest that enhancing Nrf2 function and endogenous cytoprotective mechanisms by MET, may combat age-induced ROS/RNS and protect the myocardium from oxidative stress diseases.

## Introduction

Over 75% of the mortality from cardiac diseases occurs among aged patients [1]. It has been reported that aging is an independent factor (despite hypertension, hypercholesterolemia, obesity, smoking) that accelerates oxidative stress and cardiovascular diseases [2], [3], [4], [5], [6], [7]. Therefore, it is essential to understand the mechanisms by which aging impairs redox homeostasis in the heart, paving the way to develop novel therapeutic interventions. Oxidative stress plays an important role in regulating a variety of physiological functions such as cell survival/death signaling, gene expression and various pathogenic processes [8], [9], [10], [11], [12], [13]. Previous studies have reported that aging is associated with diminished antioxidant capacity and increased accumulation of reactive oxygen and nitrogen species (ROS/RNS) [3], [6], [14], [15], [16], [17]. Sustained hemodynamic stress caused by hypertension, diabetes, and myocardial injury, excessive neurohumoral signaling and even high intensity endurance physical activity may induce pathological cardiac hypertrophy and heart failure/sudden death. The underlying molecular mechanisms for this pathology are incompletely understood and a strong need for novel therapeutic strategies remains [2], [3], [5], [6], [16], [18], [19]. However, ROS/RNS and oxidative stress are strongly associated with cardiac hypertrophy and heart failure [3], [6], [14], [15], [16], [17], [20], [21], [22].

Unexpectedly, the majority of clinical trials demonstrate that antioxidant vitamins and ROS scavengers are neither effective nor protective against cardiovascular diseases [20], [21], [23], [24]. Cellular ROS/RNS levels are regulated by the inducible defense system including antioxidant enzymes and thiol reductants. These are predominantly regulated by the transcription factor Nrf2 (nuclear erythroid-2 like factor-2) and its cytosolic repressor protein, Keap1 [25], [26], [27], [28]. Our recent work has underscored a crucial role for Nrf2 in myocardial antioxidant defense mechanisms [29]. Steady-state activation of Nrf2 provides cytoprotection from oxidant stress in cardiac myocytes. We recently observed that the Nrf2-ARE-antioxidant signaling can be activated through exercise, which represents a non-pharmacological approach to induce these antioxidant pathways [30]. Since most cardiovascular events are associated with aging, their increasing prevalence makes it essential to fully understand the mechanisms for improving endogenous cytoprotection. Some studies have reported that exercise may modulate Nrf2 signaling in rat kidney and human skeletal muscle [31], [32], [33]. However, the distinct effects of endurance exercise stress (EES) or high-intensity training (HIT) and moderate exercise training (MET) have not been addressed in the context of cardiac aging.

**Figure 1:**
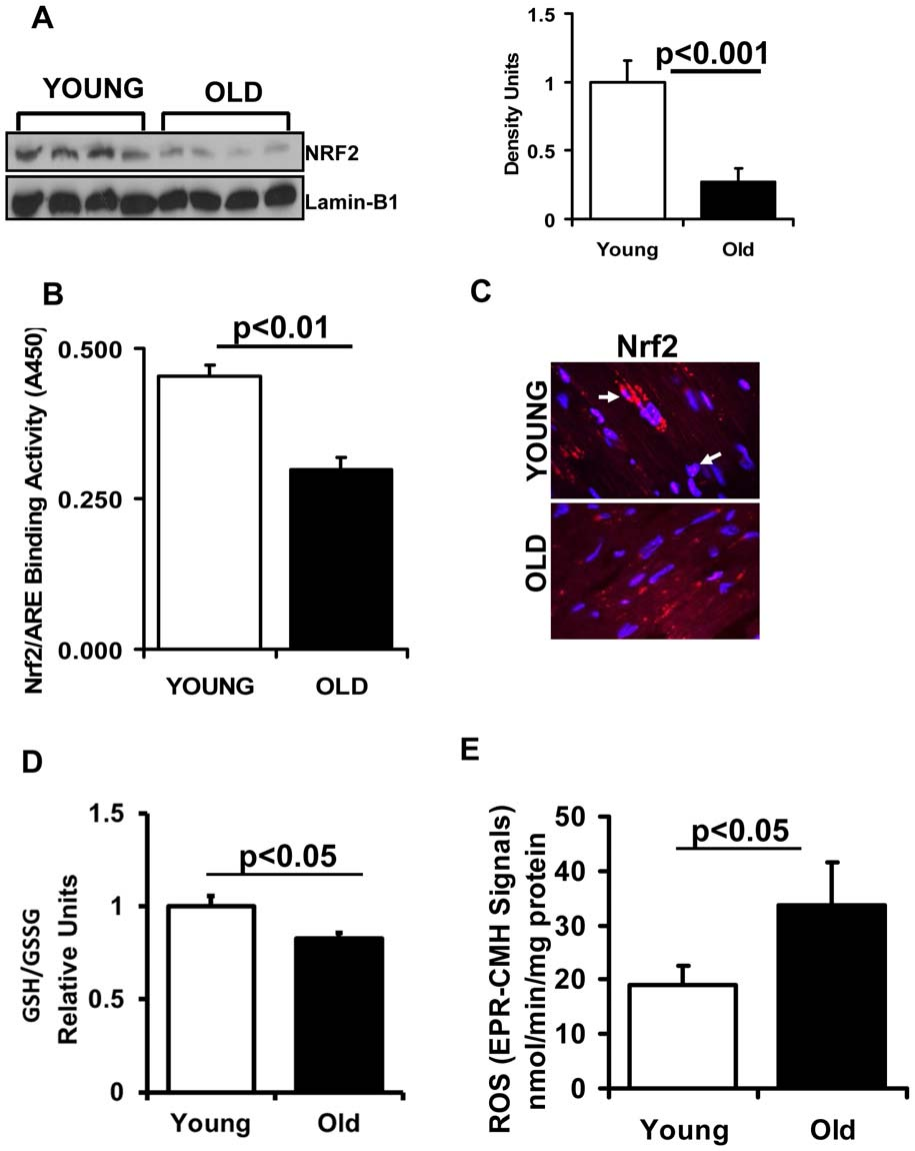
Decreased function of Nrf2 in the aging heart. (**A**) **Decreased nuclear translocation of Nrf2 is evident in the heart of aging mice.** Representative immunoblot (IB) of nuclear proteins collected from young and aged WT mice (n = 6) under basal state. Each lane represents an individual animal/heart. Lamin-B1 used as loading control. Densitometry analysis of the IB images was achieved using Image-J. (**B**) **Transcription factor binding (TransAM-Nrf2 activity) assay:** Nuclear proteins from young and aged mice (n = 4) were incubated with the oligonucleotide (pre-coated on 96 well plate/strips) for ARE (antioxidant response element). (**C**) **Localization of Nrf2 by immunofluorescence:** Immunofluorescence analysis using anti-Nrf2-ab (1∶200; v/v) showing decreased cytosolic and nuclear Nrf2 in old versus young myocardium. Blue: nucleus (DAPI); Red: Nrf2-staining and Pink: merge of blue and red indicating nuclear localization of Nrf2. IF images were obtained at a magnification of 60X oil immersion. (**D**) **Glutathione redox-state:** Myocardial redox state was determined in the ventricles of 2 and 23 months old mice under basal conditions. Statistically significant changes in the redox ratio (GSH/GSSG) were observed in young and old groups. Values are mean ± SD for 5 or more animals in each group. (**E**) **Increased reactive oxygen species (ROS) generation in the hearts of aging mice:** Electron paramagnetic resonance (EPR) spectroscopy signals for CMH (1-hydroxy-3-methoxy-carbonyl-2, 2,5,5- tetramethyl pyrrolidine) in young and aged mice. EPR signals for CMH are significantly increased in aged versus young mouse myocardium. Values represent n = 5 or more from each group.

**Figure 2:**
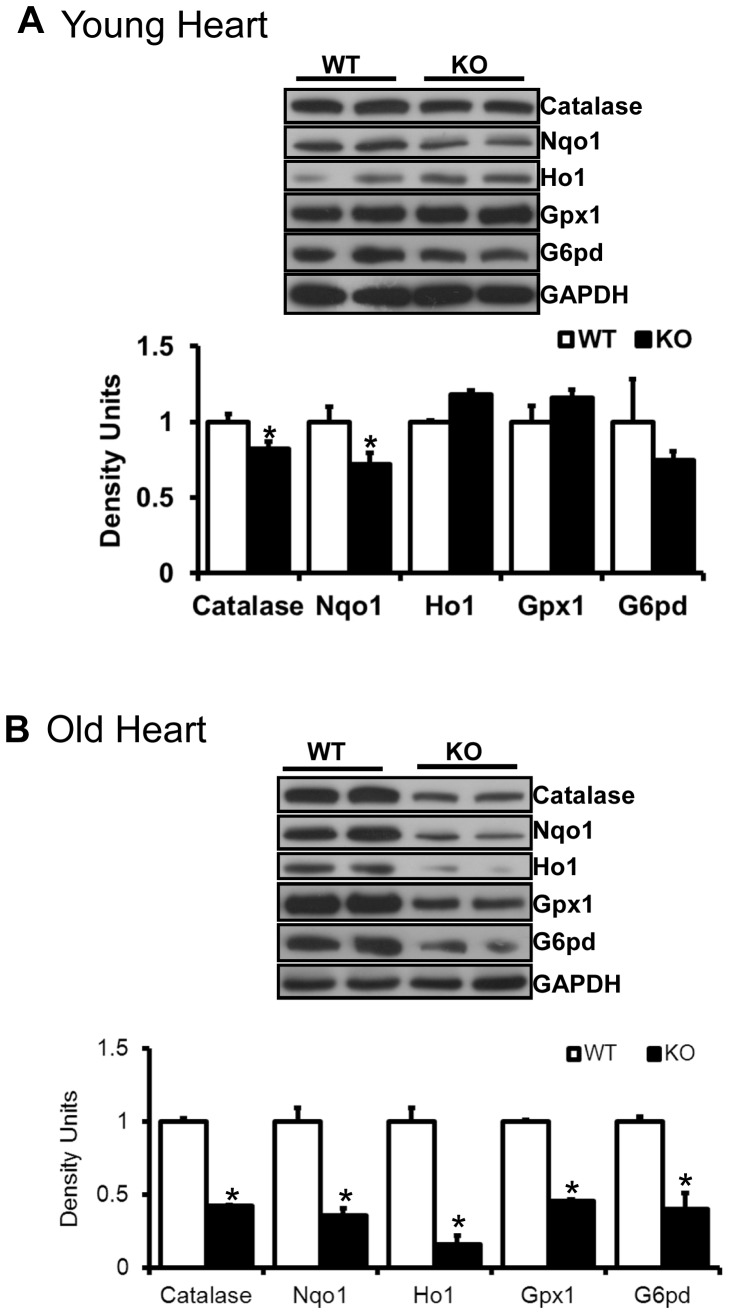
Myocardial antioxidants in young and old mice. Immunoblots showing antioxidant enzymes in hearts of young (2 months) and old (>23 months) mice. About 30 µg of cytosolic proteins from ventricle tissues were resolved on PAGE and probed for respective antibodies. Significant (p<0.05) differences were observed between WT and Nrf2-/- mice at older age (n = 4–6/group).

In this study we found that Nrf2-ARE signaling was significantly decreased along with down regulation of major antioxidant pathways in the aging heart. While acute exercise or EES triggers the Nrf2-signaling mechanisms in young mice, the aging heart was found to develop oxidative stress due to impaired levels and stability of Nrf2. In contrast, the aged mouse required prolonged and moderate exercise (6 weeks) to stabilize Nrf2/ARE-antioxidant signaling to levels that equal those observed in young mice. Thus, non-pharmacological induction of Nrf2 pathways might rejuvenate endogenous antioxidant mechanisms with resultant attenuation of age-associated oxidative stress and its related complications in the heart.

**Figure 3:**
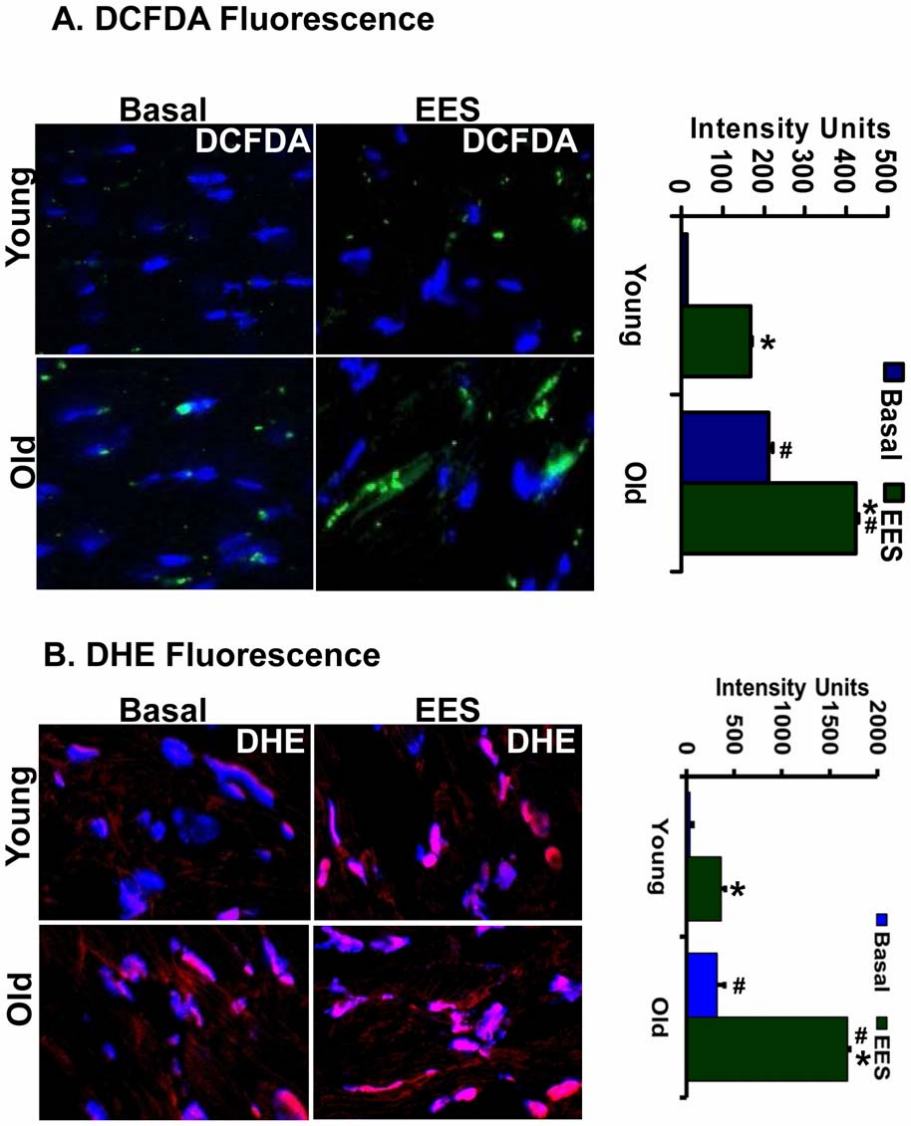
DCFDA/DHE-fluorescence in young and old mice. Myocardial tissue sections (5 µm-thick) on slides were treated with cell-permeable fluorescent H_2_DCFDA (A) and DHE (B). The microscopic images were obtained at a magnification of 60X. The oxidized, fluorescent 2-hydroxyethidium and DCF was imaged using confocal microscopy and quantified using Simple PCI 6 Imaging Software. Increased superoxide and hydrogen peroxides were observed in old heart tissues under basal and EES conditions. Green-DCF, Red-DHE and Blue-DAPI (nucleus stain). Respective intensity calculations were shown in the bar-graphs. *p<0.001- between basal and EES; #p<0.001- between young and old.

## Experimental Procedures

### Animals

Male C57/Bl6/SJ and Nrf2 knockout mice at 8–10 weeks and ≥23 months of age were used in all experiments. Breeders were either purchased from the Jackson Laboratory (Maine, USA) or obtained from Dr. Li Wang (Department of Internal Medicine, University of Utah, SLC) and maintained in our animal facility. The animals were housed 4 per cage, with access to food (standard rodent diet) and water *ad libitum* and maintained under conditions controlled for temperature and humidity, using a 12-h light/dark cycle. Both young and old wild-type (WT) mice were subjected to endurance exercise stress (EES) or moderate exercise training (MET) as described below. All experimental protocols conducted on the mice were approved by the Institutional Animal Care and Use Committee (IACUC) of the University of Utah in accordance with the standards established by the US Animal Welfare Act.

**Figure 4:**
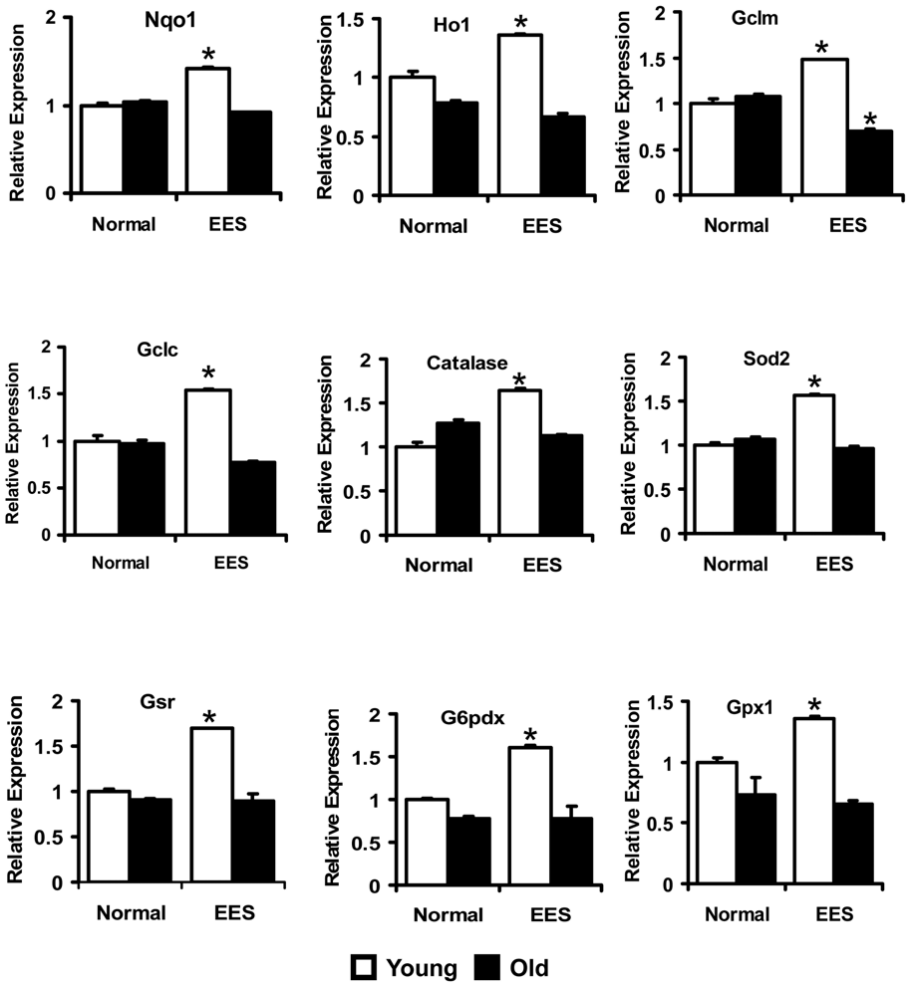
Transcription of Nrf2/ARE-dependent antioxidant genes in the heart. Real-time qPCR analyses for antioxidants were performed using Qiagen primer sets. Data were initially normalized to Arbp1 and then to the corresponding gene expression in the sedentary-young group. (n = 4–6). *p<0.05-between basal and EES.

### Antibodies and Reagents

The following antibodies and reagents were used: Nrf2-ab (SC-722, Santa Cruz Bio, SC, USA), HO-1 (ab13248, Abcam Inc., SF, USA), NQO-1 (ab34173), GAPDH (ab9485), lamin-B1 (ab16048), catalase (219010, Calbiochem, Merck kGaA, Germany), G6PD (NB100-236, Novus Biological), GCLM (ab81445), GCLC (ab40929), GCS-Ab1 (RB1697, NeoMarkers, Fremont, CA), SOD1 and SOD2 (ADI-SOD-100/110, ENZO Life Sciences). Secondary antibodies conjugated with horseradish peroxidase IgG (Rabbit and Mouse/PI-1000 & PI-2000, Vector labs, Burlingame, USA) were used. Radical detecting EPR probes – CMH (NOX-2.1), TEMPOL (705748, Sigma-Aldrich, St. Louis, MO, USA), EPR grade water (NOX-7.7.1), Krebs-HEPES buffer (NOX-7.6.1), DETC (NOX-10.1), DF (NOX-9.1), glass capillary tubes (NOX-G.3.1) and critoseal (NOX-A. 3.1-VP) were purchased from Noxygen Diagnostics, Germany. Bio-Rad Protein Assay (500-0006, Bio-Rad, Hercules, CA) was used to determine protein levels in heart tissue extracts. All reagents and primers for RNA extraction and real-time RT-PCR quantification were purchased from Qiagen Inc., Valencia, CA.

**Figure 5:**
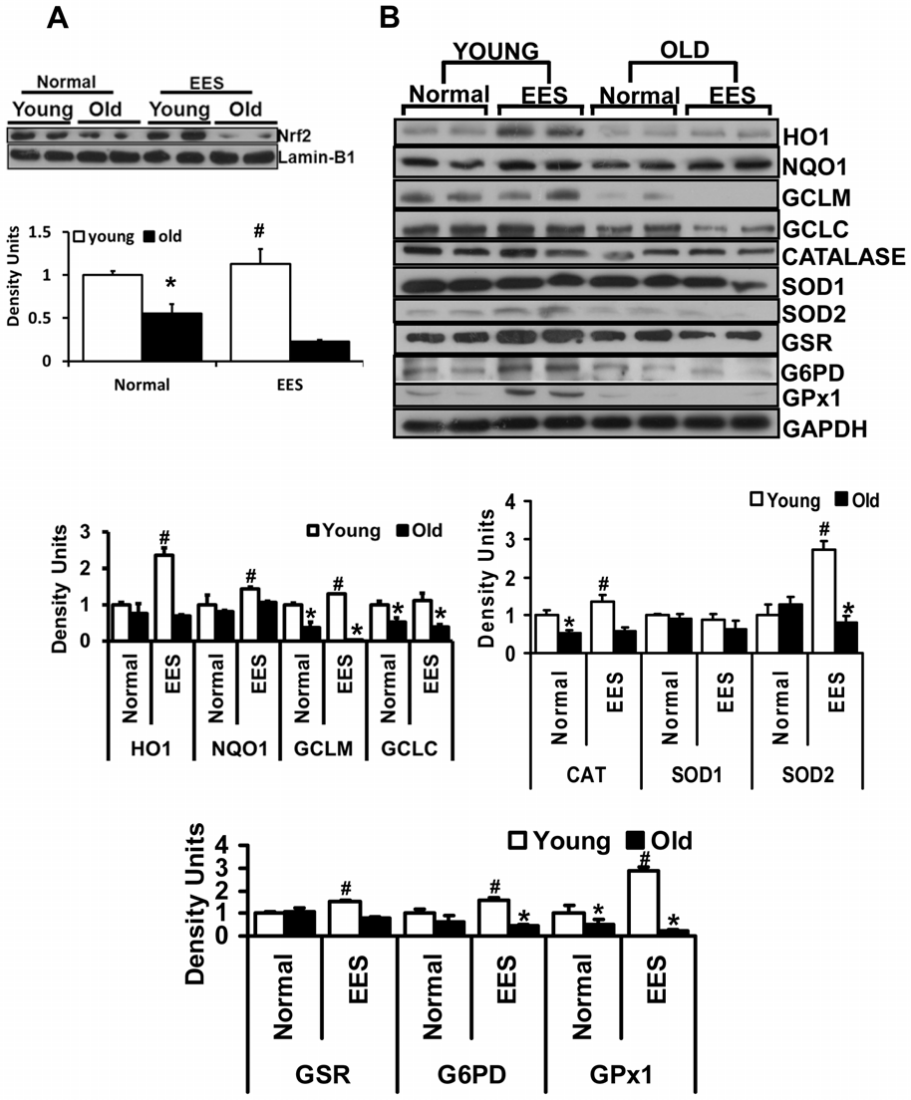
Immunoblot analyses of protein expression for Nrf2 and antioxidant enzymes. A. Analysis of nuclear Nrf2 in young and old mice subjected to EES. In sedentary mice, Nrf2 protein levels were decreased significantly in young versus old mice. EES exacerbated the decrease of nuclear Nrf2 in old mice. Blots/values represent n = 4–6 from each group. *p<0.05 between young vs. old and **^#^**p<0.05-between basal vs. EES. (B) Representative immunoblots of cytosolic extracts from the hearts of young and old mice under basal conditions and following EES. Protein blots were probed with anti-HO1, NQO1, GCLM, GCLC, Catalase, SOD1, SOD2, GSR, G6PD, GPX1 and GAPDH. Individual lanes indicate a single animal. Densitometry analysis of respective protein signals was performed using Image-J and expressed as relative intensity units calculated as mean values of young and old, *p<0.05. Individual lanes indicate each animal (n = 6). **^#^**p<0.05-between basal and EES.

### Endurance Exercise Stress (EES)

Wild type (C57/Bl6/SJ) mice at 2 (young) and >23 (old) months of age (n = 6/group/experiment) were subjected to endurance exercise on a treadmill for 2 consecutive days [∼90 minutes per day; 20m/min; 12% grade]. The duration of exercise in young mice was optimized based on the endurance ability of old mice. After exercise on the second day, mice were sacrificed and the hearts were processed/frozen (−80°C) for subsequent analysis. EPR analysis was performed in fresh heart ventricles immediately after exercise.

**Figure 6:**
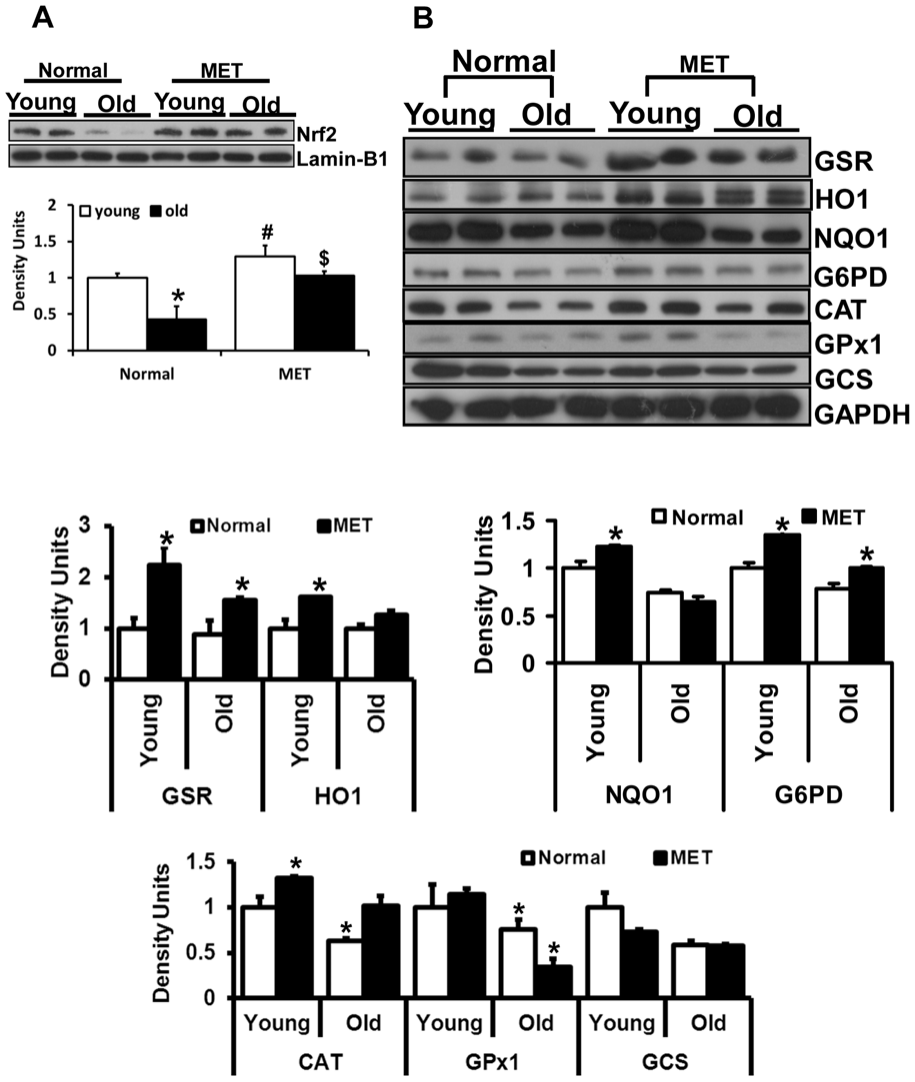
Effect of prolonged moderate exercise on Nrf2/ARE-Antioxidants in the aging heart. Representative immunoblots of cytosolic extracts from the hearts of young and old mice under basal conditions and following 6-weeks of moderate exercise training (MET). Protein blots were probed with respective antibodies as indicated. Individual lanes represent separate animals (n = 4–6/group). A. Analysis of nuclear Nrf2 in young and old mice subjected to MET. In sedentary mice, Nrf2 protein levels were decreased significantly in old when compared to young (*p<0.05). Following MET, nuclear Nrf2 levels were significantly increased in old mice to levels equivalent to those of young mice (**^#, $^**p<0.05 in MET vs. respective basal). (B) Densitometry analysis of respective protein signals were performed using Image-J and expressed relative to mean values of the sedentary-young group. Under basal conditions, a significant decrease in the protein levels of GSR, G6PD, NQO1, catalase, GPX1 and GCS were observed in the heart tissues of old when compared to young mice. Following 6-weeks of moderate exercise, most of the antioxidants were significantly (*p<0.05) upregulated or stabilized in the aging heart.

**Figure 7:**
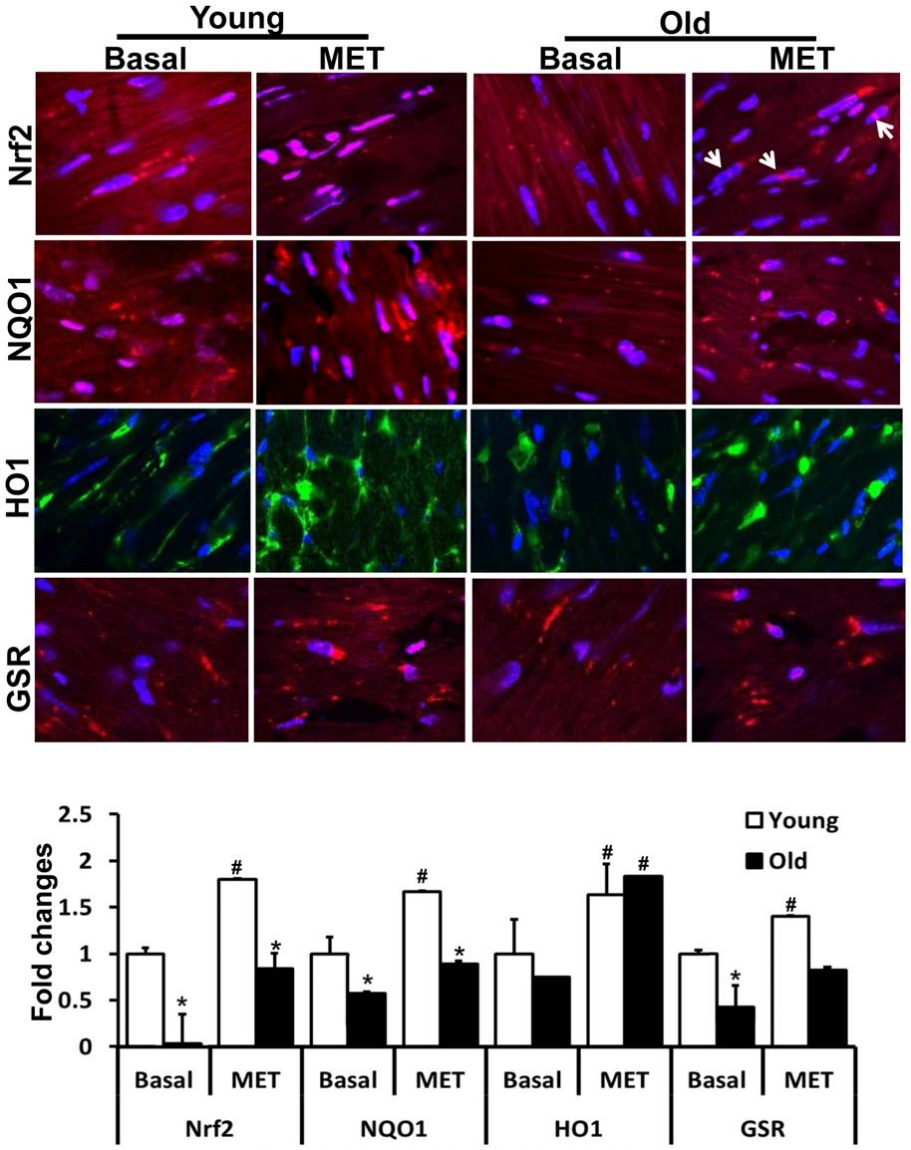
Exercise training induced Nrf2 nuclear translocation and increased antioxidant levels. Immunofluorescence (IF) analysis using anti-Nrf2-ab (1∶200; v/v) showing nuclear translocation of Nrf2 in the hearts of young and old after MET (panel 1). Blue: nucleus (DAPI); Red: Nrf2-staining and Pink: merge of blue and red indicating nuclear localization of Nrf2. Panels 2–4 depict the expression of NQO1 (1∶400), HO1 (1∶150) and GSR (1∶200) in the cardiomyocytes of young and old mice after MET. The levels of these antioxidants were comparable in the cytosol of young and old (*p<0.05) cardiomyocytes after 6-weeks of exercise training. **^#^**p<0.05 between basal and MET. IF images were obtained at 60X magnification.

### Moderate Exercise Training (MET)

To avoid stress in the aged mice, we trained them on a treadmill (50 min/day; 10m/min; 7% grade) for 6-weeks. The training protocol includes 5 minutes of ramping at 5m/min, and the speed was increased to 10m/min for 45 minutes. The mice were acclimatized to the treadmill a week before training. Sedentary control mice (n = 15) were exposed to the treadmill every day but were not exercised.

**Figure 8:**
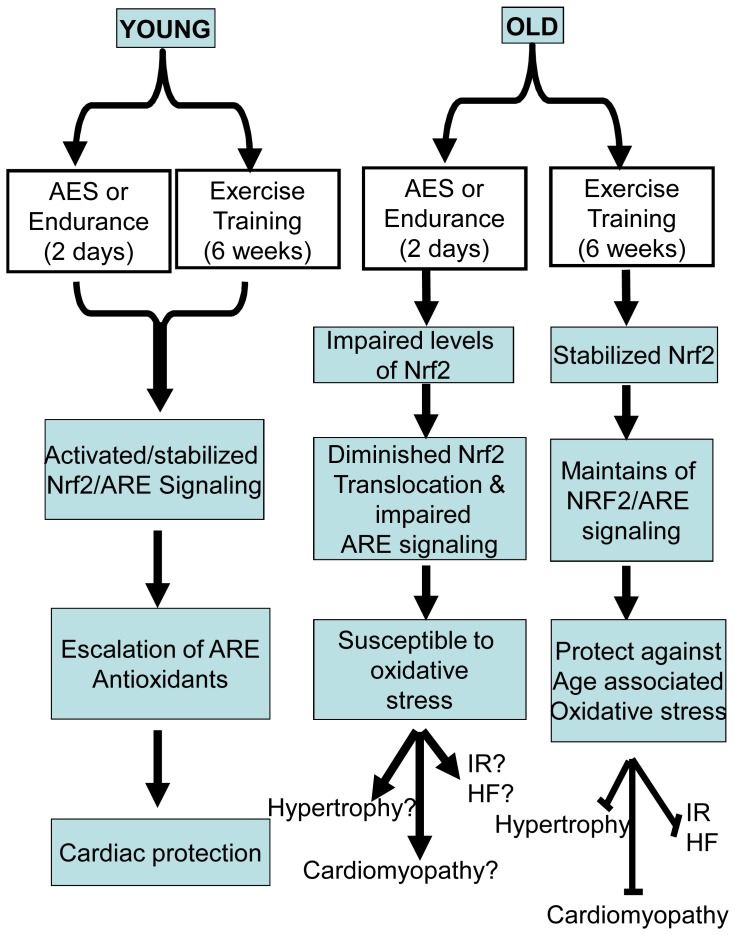
Proposed model for age-associated deregulation and exercise training mediated stabilization of Nrf2/ARE-signaling.

### Myocardial Glutathione (GSH) Redox State

Glutathione redox (GSH/GSSG) couples were measured in myocardial homogenates from young (2 months) and old (>23 months) mice. Briefly, myocardial homogenates were prepared in MES buffer (−4°C) and a known volume of supernatants were treated with equal volumes of 10% meta-phosphoric acid (MPA, Cat # 239275, Sigma) to precipitate proteins. Proteins were quantified using a small aliquot of the MES-supernatants. Tri-ethanolamine (TEAM) treated MPA-extracts were directly used for GSH and one-half of the TEAM/MPA-extracts were mixed and incubated (for 1 hr at room temperature) with 2-vinyl pyridine for GSSG analysis. A kinetic GSH-reductase recycling assay was performed following the manufacturer’s instructions (703002, Cayman Chemicals, Ann Arbor, USA) using a plate reader (Bio-Tek, FLx-800) [29], [34], [35] and calculated against appropriate GSSG standard curve.

### Determination of Reactive Oxygen Species (ROS) using Electron Paramagnetic Resonance (EPR) Spectroscopy

ROS levels in young and old myocardial tissue were determined using sensitive spin traps and EPR spectroscopy (Physics Department, University of Utah). Briefly, mice were injected (i.p.) with heparin (4 U/g b.w.) and sacrificed by CO_2_ inhalation. Hearts were perfused *in situ* with HEPES buffer (20 µM, pH 7.4) to remove blood [30]. About 10 mg of processed ventricular tissues was incubated with 150 µL of 500 µM CMH (1-hydroxy-3-methoxy-carbonyl-2, 2,5,5- tetramethyl pyrrolidine), at 37°C for 30 minutes. Nitroxide radical products of the ROS reaction with CMH was measured using an EMX-ESR spectrometer (Bruker Instruments, Germany) as reported previously [29], [30], [36].

### Detection of ROS using Fluorescent Probes (DCFDA/DHE)

Generation of superoxide and peroxides in the myocardium of young (2 months) and old (>23 months) mice under basal conditions and after EES were determined using DHE/H_2_DCFDA (D11347/C6827, Invitrogen Corp. USA) fluorescence as we previously reported [29]. The cell-permeable non-fluorescent DHE is oxidized to fluorescent 2-hydroxyethidium, which is then detected by using the excitation/emission filters appropriate for rhodamine. In the presence of superoxide (O_2_^−^•), oxidized ethidium intercalates with DNA, staining the nucleus a bright red fluorescence. Whereas hydrogen peroxide oxidizes H_2_DCFDA to a fluorescent DCF which can be detected by a fluorescent microscope using appropriate excitation/emission filters for fluorescein as reported previously [37]. Briefly, 5 µm-thick transverse sections of (optimal cutting temperature/OCT) cryo-fixed heart tissues on clean glass slides were appropriately covered with the probe solution containing DHE/H_2_DCFDA (10 µM) and were incubated in a light-protected chamber at 37°C for 30 min, washed thoroughly with 1X PBS-T thrice, fixed and mounted using DAPI containing Vector-Flouromount -G. Fluorescent images of the DCF and DHE were obtained using a FV-1000 laser scanning confocal microscope (Olympus Inc.). Fluorescence intensity was quantified by automated image analysis using the Simple PCI 6 Imaging Software (Hamamatsu Corporation, Sewickley, PA).

### Determination of Nrf2-antioxidant Response Element (ARE) Binding Efficiency

The levels of Nrf2-ARE binding in young and old mouse hearts were measured in nuclear proteins using a Trans AM Nrf2 Kit (50296, Active Motif, Carlsbad, CA). Ten µg of nuclear protein was incubated in 96-well plates pre-coated with ARE consensus oligonucleotides (5`-GTCACAGTACTCAGCAGAATCTG-3`), and the active-Nrf2 that bound to the oligo was detected by incubating with anti-Nrf2-ab and appropriate HRP-conjugated secondary antibody. The final product formed as a result of specific activity of the transcription factor in the nuclear extracts was read using a plate reader at 450 nm, and absorbance was expressed as the direct activity of Nrf2.

### Western Blotting and Quantification of Myocardial Antioxidant Enzymes

Cytosolic and nuclear proteins were extracted from blood-free (heparin injected and buffer perfused *in situ*) hearts of young and old mice [29]. First, using homogenizing buffer [10 mM HEPES, 10 mM KCl, 0.1 mM EDTA, 0.5 mM MgCl**_2_**, with freshly prepared 1 mM dithiothreitol and 0.1 mM phenyl methylsulfonyl fluoride (PMSF) and 1% Triton X100, pH 7.9], cytosol was fractionated through centrifugation at 5200 rpm for 5–6 minutes. The pellet (nucleus) was washed with 4 volumes of homogenization buffer to remove the residual cytosolic proteins. Next, nuclear proteins were prepared by incubating the pellets in complete lysis buffer (20 mM HEPES, 420 mM NaCl, 0.1 mM EDTA, 1.5 mM MgCl**_2_**, 25% glycerol and 1 mM dithiotheitol, 0.5 mM PMSF, pH 7.9) and centrifugation at 8200 rpm for 10 minutes. Protein quantification and immunoblotting for major antioxidants including catalase, SOD, GPX1, GSR, G6PD, HO-1, NQO-1, ã-GCS, GCLM, GCLC and GAPDH were performed using standard protocols as described in our previous reports [29], [30], [34]. For determining Nrf2 levels in young and aged mice, ∼40 µg of nuclear proteins were separated on 10% SDS-PAGE and probed against Nrf2-ab and lamin-B1 as loading control. Appropriate secondary antibodies (rabbit and mouse) conjugated with HRP-IgG were used for chemiluminescence detection of blotted proteins. Density of the protein bands were analyzed using the NIH Image-J program.

### Isolation of RNA, cDNA Synthesis and qPCR Analysis of Antioxidant Genes

Total RNA was purified from young and old mouse hearts that were harvested after an *in situ* perfusion with 10 ml of RNase free PBS and 10 ml of RNA later reagent. About 30 mg of ventricular tissues were processed using RNA extraction kits (Qiagen #74106) following the supplier’s instructions. Nano-drop measurements of RNA samples were processed to determine quality and quantity of the RNA. Using 2.5 µg RNA from each heart, cDNA was synthesized as per the protocols described in Qiagen Reverse Transcription Kit (Cat# 205311). For qPCR analysis, 100 ng of cDNA template, 10 µL of SYBR green master mix (Qiagen # 204054) and respective Qiagen primer sets for Ho-1 (QT00095270), Nqo-1 (QT00094367), catalase (QT01058106), Sod1 (QT01762719), Sod2 (QT00161707), G6pdx (QT00120750), Gclc (QT00130543), Gclm (QT00174300), Gsr (QT01758232) and Gpx1 (QT01195936) were used and analyzed in a Light Cycler real-time thermocycler (Roche Bio). Copy numbers of cDNA targets were quantified using Ct values, and the mRNA expression levels for all samples were normalized to the level of the housekeeping gene Arbp1 (QT00249375) or Gapdh (QT01658692).

### Localization of Nrf2 and Other Antioxidant Enzymes by Immunofluorescence (IF) Analysis

Frozen (OCT) myocardial sections at 5 µm thickness from the sedentary and exercised C57/Bl6 mice at 2 and 23 months old were immunostained with respective primary antibodies as reported previously [29], [34]. The tissue sections were fixed in 4.0% paraformaldehyde for 10 minutes, permeabilized with 0.25% Triton X-100 in PBS containing 0.01% Tween-20 for 5 min, blocked for 60 min with 5% goat serum in 0.01% PBST and incubated with appropriate primary antibody diluted in 0.01% PBST containing 1% BSA over night at 4°C. After 3X10 min washes with PBST, the sections were incubated with appropriate secondary anti-mouse Alexa-Fluor 488-conjugated or anti-Rabbit Alexa-Fluor 647-conjugated antibodies (1∶1000 dilution) for 1 hour at room temperature. After 3 washes (5 minutes each) with 1X PBST, the sections were mounted with DAPI containing mounting medium (Vector Shield) and imaged using FV-1000 Confocal (Olympus Inc.) microscope at a magnification of 60X for visualization and specific localization of NRF2 (1∶200), NQO1 (1∶400), HO1 (1∶150) and GSR (1∶200).

### Statistical Analysis

Data were normalized to the respective control mean values and are expressed as mean ± SD for n = 5 or more animals in each experimental group. Statistical analysis of the data was performed by Student’s *t* test. Unless otherwise indicated, a P value of <0.05 was considered statistically significant.

## Results

### Nrf2 Signaling is Impaired in the Aging Heart

We recently reported that the Nrf2-Keap1 pathway is critical for myocardial antioxidant defense function [29], [30]. In the current investigation, we report on the effect of aging on this pathway. Western blot analysis revealed a significant decrease of Nrf2 (1.0±0.18 vs. 0.35±0.12; young vs. old) in the nuclear extracts of myocardium isolated from aged (>23 months) versus young (2 months) mice (Fig. 1A). Nrf2-ARE-binding efficacy was significantly reduced (0.6 fold) in the aging heart (Fig. 1B). To further confirm these results, immunofluorescence analysis using anti-Nrf2-ab revealed decreased cytosolic and nuclear Nrf2 in old versus young myocardium (Fig. 1C). These results indicate age-associated down regulation of Nrf2 signaling in the heart. Interestingly, abrogation of Nrf2 resulted in a moderate decrease of some myocardial antioxidants (catalase and NQO1) in young Nrf2-/- when compared to WT mice (Fig. 2A). However, with aging, Nrf2-/- mice had significant (p<0.05) down regulation of most of the Nrf2 targets including G6PD, NQO1, catalase, HO1and GPX1 (Fig. 2B). Taken together, the results demonstrate an age-associated impairment of antioxidant pathways in the heart that is exacerbated by absence of Nrf2/ARE signaling.

### Impaired Nrf2 Signaling is Associated with GSH Depletion and Oxidative Stress in the Aging Heart

To assess whether the loss of Nrf2 affects intracellular redox homeostasis, we measured ROS levels and the glutathione redox state. Significantly decreased GSH was observed in the hearts of aging versus young mice (Fig. 1D). In association with the impaired redox state, there was a significant increase of ROS levels in the aging heart when measured by EPR (Fig. 1E) or fluorescent probes (Fig. 3A-B), suggesting age-associated oxidative stress due to impaired Nrf2/ARE-signaling. In particular, significantly increased superoxide (DHE) and hydroperoxides (DCF) were observed in old versus young myocardial tissue (Fig. 3). Since aging hearts exhibit oxidative stress, we then tested the hypothesis that EES might exacerbate this condition in the aging heart. ROS levels were increased after EES in both young and old mice when compared to the respective sedentary groups. Moreover, the aging myocardium had increased superoxide and hydroperoxides relative to young mice following EES (Fig. 3A-B).

### Endurance Exercise Stress Down Regulates Antioxidant Genes in the Aging Heart

Several reports indicate that Nrf2 targets over 100 genes that are potentially regulated through ARE in their promoters [28], [38], [39], [40], [41]. We hypothesized that the age-associated decrease in nuclear content and ARE-binding activity of Nrf2 (Fig. 1) might suppress transcriptional activation of downstream targets, including genes that are involved in the production of intracellular reducing equivalents such as glutathione and NADPH. To evaluate this hypothesis, we performed qPCR analysis using cDNA synthesized from RNA of young and aged mouse hearts. These analyses indicate repression or impaired induction of most of the antioxidant genes evaluated in aged compared to young myocardium following EES. The major targets of Nrf2 such as Nqo1 and Ho1 were either decreased or their induction was abolished in the hearts of aged mice when compared to young mice following EES (Fig. 4). Genes encoding for the sub-units of ã-glutamyl cysteine ligase (GCL), the rate limiting enzyme for GSH biosynthesis, Gclm and Gclc were also significantly repressed in the aging heart when compared to young mice following EES (Fig. 4). Genes encoding antioxidant enzymes that involve ROS scavenging such as Sod2, catalase and Gpx1 were also differentially regulated with aging. Although mRNA levels for Sod2, catalase and Gpx1 were significantly induced in the heart of young mice following EES, induction of stress-mediated genes was blunted in the old mice. Similarly, induction of mRNA levels for G6PD and GSR, key enzymes responsible for recycling oxidized glutathione (GSSG) back into its reduced form (GSH), exhibited a similar trend, being increased in young, but blunted in old mice following EES. These results support the hypothesis that absence of Nrf2 signaling pathway might be responsible for impairing the transcription of these genes.

### Endurance Exercise Stress (EES) Exacerbate Dysfunction of Nrf2/ARE-antioxidant Signaling in the Aging Heart

We next determined nuclear Nrf2 levels in young and aged mice subjected to EES. Nuclear Nrf2 levels were significantly decreased in aged relative to young mouse myocardium. A significant increase in nuclear Nrf2 levels was observed in the hearts of young mice following EES (Fig. 5A). To assess whether the loss of Nrf2 was coupled with impaired protein synthesis of major antioxidant enzymes that are involved in glutathione/NADPH metabolism and ROS scavenging, we performed Western blots using cytoplasm of young and aged hearts. Protein levels for NQO1, HO1, GCLM, GCLC, catalase, SOD2, G6PD and GPX1 were significantly decreased in the hearts of aged relative to young mice (Fig. 5B). These results parallel changes in gene expression indicating tight regulation of their transcription through Nrf2 signaling, which is impaired in the aging heart following EES (Fig. 5A). Of note, there were no distinguishable changes in protein expression for GSR and SOD1 between the young and aged myocardium. It is conceivable that upon age-associated diminished Nrf2/ARE signaling, there might be an increase in recruitment of antioxidants to cope with oxidative stress in the aging heart. However, dramatic decrease in the majority of the antioxidant enzymes for a prolonged period might likely contribute to developing oxidative stress diseases in the aging myocardium. Next, to test the susceptibility of age-dependent Nrf2 dysfunction, we subjected the mice to endurance exercise stress (EES) and evaluated the myocardial defense system. While most of the antioxidants (NQO1, HO1, catalase, GSR and GPX1) were significantly upregulated in the heart of young mice; the aging myocardium had depleted antioxidant enzymes following EES (Fig.5B). These results suggest that aged mice could not adapt to EES due to accelerated utilization of the antioxidant pool to overcome the stress.

### Prolonged and Moderate Intensity Exercise Training (MET) Increases the Nrf2-Antioxidant Enzymes in the Aging Mouse

We hypothesized that in contrasts to EES, moderate exercise training (MET) for an extended period of time would enhance Nrf2 signaling and thereby increase its target antioxidants in the aging heart. To test this hypothesis, we performed western blots and immunofluorescence analysis in young and aged mice trained for prolonged, but moderate, exercise for 6-weeks to produce a physiological and sustained induction of Nrf2/ARE signaling (Fig. 6A and 7). In response to MET, nuclear Nrf2 protein was significantly increased in the hearts of both young and old mice, suggesting that moderate training promotes stability of Nrf2 and thereby enhances transactivation of target antioxidants (Fig. 6A). As expected, protein levels for major antioxidant enzymes were significantly increased in the aging heart after 6-weeks of MET when compared to the sedentary group. Although, the exercise-mediated induction of antioxidants in aged mice was not as robust as that seen in the younger group, they were significantly increased relative to the sedentary-aged group (Fig. 6B). The level of nuclear Nrf2 was much higher in young as compared to old mice. NQO1, HO1 and GSR (Fig. 7) levels were comparable in the cytosol of young and old cardiomyocytes after 6-weeks of MET. Taken together, these observations support the notion that increased Nrf2 signaling may induce its major target antioxidant enzymes in response to prolonged moderate intensity exercise training in the aging myocardium.

## Discussion

Results from the current investigation reveal that (i) myocardial Nrf2 expression declines with age, (ii) endurance exercise stress (EES) or high intensity training (HIT) promotes Nrf2-signaling in young, but paradoxically diminishes major antioxidant enzyme levels in aging mice, (iii) prolonged MET stabilizes Nrf2-signaling and improves ARE-dependent antioxidants in the aging myocardium. Thus, prolonged MET represents a potent non-pharmacological inducer of endogenous antioxidant pathways. While moderate exercise training is regarded as an essential mode of prevention and/or therapy for cardiovascular health, the relative effect of intensive endurance or physical high intensity versus moderate intensity exercise is incompletely understood. Previous studies have documented that chronic pathological conditions including cardiovascular diseases (CVD) may be prevented by moderate physical activity [42], [43]. On the other hand, a strong negative correlation has been postulated between aging and antioxidant efficacy, via incompletely understood mechanisms. We recently showed that acute exercise stress (AES) promotes antioxidant pathways via Nrf2-signaling in the young mouse [30]. There is substantial evidence that Nrf2 plays an important role in regulating basal and inducible transcription of most antioxidant and cytoprotective genes in the heart [26], [28], [38], [39], [40], [44]. However, only a few studies have examined the role of exercise training in the regulation of Nrf2 [29], [30], [45], [46], [47]. A major finding presented in the current investigation is that sustained MET in the aging myocardium augments endogenous antioxidant mechanisms through Nrf2.

### Aging Impairs Nrf2-signaling and Intracellular Redox State in the Myocardium

Prior studies indicate that nuclear translocation/stability and function of Nrf2 in blood vessels, skeletal muscle and liver are decreased in association with aging [14], [33], [48], [49]. Our recent findings reveal that sustained increases in nuclear Nrf2 levels increase glutathione (GSH) content along with upregulation of major antioxidant genes in the heart [29]. However, the correlation between functional Nrf2 and the antioxidant system were not addressed in the aging heart. The present study demonstrates that depletion of myocardial GSH correlates with diminished nuclear Nrf2 levels in the aging mouse heart. As a consequence of impaired antioxidant mechanisms, the aging mouse heart has increased ROS content as determined by the EPR and redox sensitive fluorescent probes. AES induces Nrf2 nuclear translocation and subsequent upregulation of transcriptional machinery which is responsible for GSH metabolism in young wild-type (C57/Bl6) mice, whereas Nrf2-/- mouse hearts had insufficient GSH and exhibited severe oxidative stress upon AES [30]. The diminished GSH could result from two mechanisms: (i) via direct reaction of GSH with oxidants formed during aging or (ii) due to decreased production of GSH in association with impaired Nrf2-signaling. In the aging myocardium, this impaired redox homeostasis resulted in age-associated enhanced oxidative stress. We speculate that depletion of GSH resultant increased oxidative stress with aging accelerate susceptibility to myocardial damage and cardiac disease. Thus the age dependent regulation of Nrf2 may play a critical role for maintaining redox homeostasis in the heart.

### Aging is Associated with Insufficient Production of ARE-dependent Antioxidants in the Heart

To our knowledge, the present investigation represents the first detailed characterization of myocardial Nrf2-ARE redox signaling in the context of aging-mediated oxidative stress. Recent studies demonstrate that Nrf2 enhances tolerance to oxidative stress and increases lifespan of *Drosophila* and proliferation of intestinal stem cells by promoting redox homeostasis [50], [51], [52]. In the current study, we demonstrate that aging is associated with diminished transcription of many antioxidant genes. Nrf2 is critical for the transcription of antioxidant response element (ARE) containing genes, but loss of Nrf2 also may affect their trans-activation [53]. Though young mice exhibited compensatory trans-activation of ARE-containing antioxidant genes, aged mice had increased ROS generation following EES. Protein levels for most of the antioxidants (HO1, GCLM, GCLC, Catalase, G6PD and GPX1) were significantly decreased in aged mice under basal conditions and these mice exhibited poor compensatory response to EES relative to young animals. A decrease in ARE-transcriptional machinery in the myocardium might be closely associated with age-dependent accumulation of ROS and chronic oxidative stress. A significant decrease of myocardial Nrf2 was observed in older (>23 months) mice when compared with young (∼2 months) mice, as previously shown in the rat liver [48]. In response to aging, though we observe decreased levels of Nrf2 and its interaction with the ARE in old mice when compared to the young animals, it is uncertain why Nrf2 is not activated to a comparable level observed in the young hearts despite increased levels of ROS in the aged mouse. This raises the possibilities of diminished synthesis, stabilization or half-life of the Nrf2 upon aging. In the unstressed state, the aging heart maintains normal function despite a moderate decrease in nuclear Nrf2, but is unable to maintain redox homeostasis in response to EES. Further investigation is required to validate whether EES could increase the susceptibility of the aging myocardium to challenges such as ischemia/reperfusion (I/R) injury or pathological cardiac hypertrophy induced by TNF-á or angiotensin-II administration.

### Endurance Exercise Stress (EES) Exacerbates Deregulation of Nrf2-antioxidant Mechanisms upon Aging

Accumulation of ROS/RNS likely leads to increased susceptibility of the aging myocardium to oxidative damage. Though moderate exercise has been reported to protect the myocardium [54], [55], [56], [57], our findings indicate that endurance exercise stress (EES) actually depletes antioxidants via Nrf2 dysfunction in the aging heart. Moreover, the age-dependent increase in ROS was further exacerbated by EES-mediated depletion of Nrf2 antioxidants. We observed that while young mice exhibited compensatory induction of antioxidant genes and enzymes, old mice developed severe oxidative stress in response to EES. Recent studies report that acute intense endurance exercise may lead to right ventricular cardiac dysfunction and cardiomyopathy in athletes, but these abnormalities were minimal in high intensity trained athletes [58], [59]. It has also been proposed that limited pre-event training may increase the risk of cardiac injury after an endurance marathon [60], [61]. Another study indicates that endurance exercise promotes hematopoiesis and increased cytokine production in skeletal muscle [62]. These observations suggest distinct effects for high intensity training that are unique to distinct cell types. Here we demonstrate that EES impairs myocardial antioxidants through diminished Nrf2/ARE-signaling, but moderate exercise training for extended durations facilitates Nrf2-dependent cytoprotective mechanisms in the aging myocardium.

### Moderate Exercise Training is Necessary and Sufficient to Promote Endogenous Nrf2/ARE-antioxidant Mechanisms in Aging Myocardium

Given the important regulatory role played by Nrf2 and aging on antioxidant protection in the heart, the data presented herein suggests that moderate intensity exercise training, rather than endurance stress, enhances nuclear levels of Nrf2 (Fig. 6A) and induces key antioxidant enzymes in the aging heart (Fig. 6B & 7). It has been reported that sedentary older humans exhibit Nrf2-Keap1 dysfunction, but an active life style increases Nrf2 function and thereby maintains redox homeostasis in skeletal muscles of older humans [33]. In the current study, since the endurance stress impairs Nrf2-signaling and antioxidants in aging myocardium. We speculated that subjecting aged mice to moderate exercise training for a prolonged duration (6 weeks) might lead to an adaptive response by stabilizing Nrf2 signaling. Analysis of Nrf2-dependent antioxidant mechanisms after 6-weeks of MET revealed significant and comparable nuclear Nrf2 levels in the cardiomyocytes (as shown in IF analysis, Fig. 7) of young and old mice. Further, this activation of Nrf2 following MET can be attributed to a marked increase in antioxidant enzymes such as GSR, HO1, G6PD, catalase, GPX1 and GCS in the myocardium of young and old mice.

### Conclusion

Increasing adaptive cytoprotective response in an aging heart can be achieved through moderate exercise training. Based on our findings, it is expected that non-pharmacological induction of Nrf2 pathway enhances endogenous antioxidant mechanisms that could prevent age-associated oxidative stress complications in the heart. While the inducible antioxidant system is activated in young mice, endurance exercise stress significantly impairs Nrf2-antioxidant pathways in aging mouse hearts. Our studies provide evidence, for the first time, that moderate exercise training for a prolonged duration prevents age-associated Nrf2 dysfunction and thereby increases the ability of myocardium to cope with enhanced oxidative stress (Fig. 8). Future therapeutic protocols may be targeted using Nrf2 for manipulating redox stress conditions in aging myocardium. Nrf2 can be activated pharmacologically by small molecules [63], [64], [65], [66], providing an additional means to attenuate myocardial oxidative stress that occurs with aging. Thus, our immediate future goals include determining whether spontaneous chronic exercise and small molecules can induce Nrf2-signaling and confer cardio-protection in the aging heart.
