# Correction: Impaired Transcriptional Activity of Nrf2 in Age-Related Myocardial Oxidative Stress Is Reversible by Moderate Exercise Training

**DOI:** 10.1371/annotation/8690bb36-3c5d-48a6-b3be-39a2b50896e1

**Published:** 2012-10-03

**Authors:** Sellamuthu S. Gounder, Sankaranarayanan Kannan, Dinesh Devadoss, Corey J. Miller, Kevin J. Whitehead, Shannon J. Odelberg, Matthew A. Firpo, Robert Paine, John R. Hoidal, E. Dale Abel, Namakkal S. Rajasekaran

There were two errors in the byline. The equal contributions designation was missing. Authors Sellamuthu S. Gounder and Sankaranarayanan Kannan contributed equally to this work. Also, the fifth author's name was incorrect. Kevin J. Whitehead is the correct name. The correct citation is:

Gounder SS, Kannan S, Devadoss D, Miller CJ, Whitehead KJ, et al. (2012) Impaired Transcriptional Activity of Nrf2 in Age-Related Myocardial Oxidative Stress Is Reversible by Moderate Exercise Training. PLoS ONE 7(9): e45697. doi:10.1371/journal.pone.0045697 

