# Correction: Impaired Transcriptional Activity of Nrf2 in Age-Related Myocardial Oxidative Stress Is Reversible by Moderate Exercise Training

**DOI:** 10.1371/annotation/684807c2-1777-4008-9eda-c6036553b082

**Published:** 2013-06-24

**Authors:** Sellamuthu S. Gounder, Sankaranarayanan Kannan, Dinesh Devadoss, Corey J. Miller, Kevin S. Whitehead, Shannon J. Odelberg, Matthew A. Firpo, Robert Paine, John R. Hoidal, E. Dale Abel, Namakkal S. Rajasekaran

There is a funder and grant number missing from the Funding Statement. The following is the missing information:

This work was also supported by the the National Institute of Aging (NIA), with the Grant #1R03AG042860-01. 

